# *Streptococcus dysgalactiae* Bloodstream Infections, Norway, 1999–2021

**DOI:** 10.3201/eid2902.221218

**Published:** 2023-02

**Authors:** Oddvar Oppegaard, Marte Glambek, Dag Harald Skutlaberg, Steinar Skrede, Audun Sivertsen, Bård Reiakvam Kittang

**Affiliations:** Haukeland University Hospital, Bergen Norway (O. Oppegaard, M. Glambek, D.H. Skutlaberg, S. Skrede, A. Sivertsen);; University of Bergen, Bergen (O. Oppegaard, S. Skrede, B.R. Kittang);; Haraldsplass Deaconess Hospital, Bergen (B.R. Kittang)

**Keywords:** Streptococcus dysgalactiae, bacteria, streptococci, beta-hemolytic streptococcus, bloodstream infections, epidemiology, incidence rates, Norway

## Abstract

*Streptococcus dysgalactiae* increasingly is recognized as a pathogen of concern for human health. However, longitudinal surveillance data describing temporal trends of *S. dysgalactiae* are scarce. We retrospectively identified all β-hemolytic streptococcal bloodstream infections reported in Bergen, in western Norway, during 1999–2021. To explore *S. dysgalactiae* disease burden in a broader context, we mapped the incidence of all microbial species causing bloodstream infections during 2012–2021. We found *S. dysgalactiae* incidence rates substantially increased during the study period; by 2021, *S. dysgalactiae* was the fifth most common pathogen causing bloodstream infections in our region. We noted genotypic shifts and found that the rising trend was related in part to the introduction and expansion of the *stG62647 emm*-type. *S. dysgalactiae* is among the most common causes of bloodstream infections in western Norway, and increased surveillance and unambiguous species identification are needed to monitor the disease burden attributable to this pathogen.

*Streptococcus dysgalactiae* is a potent pathogen that has similar disease manifestations as *S. pyogenes*, including cellulitis, necrotizing soft tissue infections, and streptococcal toxic shock syndrome (*1*–*3*). Nevertheless, in contrast to the well-established surveillance networks for *S. pyogenes* and *S. agalactiae* infections in many countries, epidemiologic reports on *S. dysgalactiae* are scarce (*4*–*6*).

The *S. dysgalactiae* epithet and delineation of its 2 subspecies, *dysgalactiae* and *equisimilis*, was proposed as late as 1996, and until recently identification of this pathogen has relied predominantly on phenotypic β-hemolysis and a Lancefield agglutination reaction (*7*). However, the promiscuous repertoire of Lancefield antigens potentially displayed by *S. dysgalactiae* (mostly C or G, occasionally A or L) has caused considerable taxonomic confusion (*8*). In addition, *S. equi* harbors the C antigen and *S. canis* harbors the G antigen, which adds further ambiguity to speciation. Although *S. equi* and *S. canis* rarely cause human disease, their added ambiguity further illustrates that the Lancefield classification lacks taxonomic resolution to the species level for the group C and G *Streptococcus* (GCGS).

Clinical and epidemiologic features of *S. dysgalactiae* have often been fragmented and concealed in separate scientific reports on group C and group G *Streptococcus*, a practice that continues to some extent (*9*–*12*). This practice has likely underestimated *S. dysgalactiae* incidence rates and clouded its clinical significance. Moreover, most recent publications involving precise identification of *S. dysgalactiae* comprise insufficient sample sizes or observation periods to evaluate temporal trends (*3*,*13*–*15*).

Drawing power from the enhanced taxonomic precision of sequencing technology and mass spectrometry, we revisited *S. dysgalactiae* bloodstream infection epidemiology in a health region in western Norway during the past 23 years. Moreover, to explore *S. dysgalactiae* disease burden in a broader context, we compared *S. dysgalactiae* incidence against all other pathogens that cause bloodstream infections. 

## Methods

### Study Setting and Definitions

Health Region Bergen (Bergen, Norway) had a catchment area of ≈460,000 inhabitants in 2021 and encompasses the tertiary care institution Haukeland University Hospital and 2 local hospitals, Haraldsplass Deaconess Hospital and Voss Hospital. The region is served by a single microbiology laboratory located at Haukeland University Hospital.

We reviewed the electronic records of the microbiology laboratory and retrospectively identified all bloodstream infections caused by *S. pyogenes*, *S. agalactiae*, and *S. dysgalactiae* in Health Region Bergen during 1999–2021. We also extracted information on specimen type, sample date and species identification, as well as patient age, sex, and residential postal code.

We defined bloodstream infection as microbial growth in blood cultures collected from patients in a hospital setting. We considered cases from which repeated cultures were positive for the same identified organism within 30 days of initial isolation as a single bloodstream infection. To avoid referral bias, we excluded patients with a residential postal code outside the catchment areas of the 3 hospitals at the time of specimen sampling.

To explore the incidence of *S. dysgalactiae* bloodstream infections in a wider context, we also retrospectively identified bloodstream infections caused by other pathogens during 2012–2021 in our region. We confined the study period to a decade that had relatively stable standards and protocols for pathogen identification in the routine microbiology laboratory. The main tool for species identification during this era was matrix-assisted laser desorption/ionization time-of-flight (MALDI-TOF) mass spectrometry and the MALDI Biotyper database (Bruker Daltonik, https://www.bruker.com), which received annual or biannual updates from the manufacturer.

### Bacterial Identification of β-hemolytic Streptococci

Primary identification in the routine microbiology laboratory was based on β-hemolytic reaction on 5% sheep-blood agar, colony size of >0.5 mm after 24 h incubation, and Lancefield serogroup specificity (Oxoid, http://www.oxoid.com). Identification of β-hemolytic streptococci was supplemented with MALDI-TOF mass spectrometry beginning in 2012. For this study, we retrieved all β-hemolytic streptococcal isolates identified in bloodstream infections during 1999–2021 from the freezer and confirmed species identity by MALDI-TOF mass spectrometry. Moreover, we taxonomically verified all strains identified as *S. dysgalactiae*, *S. canis*, *S. equi*, or *S. pyogenes* by using either *emm* typing (*16*) or whole genome sequencing (*17*), as previously described. A subset of these strains had already been characterized by *emm* typing in previous studies (*1*,*17*).

### Statistical Analyses

We calculated incidence rates by using contemporary population data extracted from Statistics Norway (https://www.ssb.no). We tested temporal trends for statistical significance by using negative binomial regression analysis and evaluated nonparametric data by using the Mann Whitney U-test. We considered a 2-sided p value <0.05 statistically significant. We used SPSS Statistics 26.0 (IBM, https://www.ibm.com) for statistical analyses, Excel 2016 (Microsoft, https://www.crosoft.com) to create graphs, and R version 4.1.2 (The R Foundation for Statistical Computing, https://www.r-project.org) to construct violin plots. Regional Ethics Committee West, Norway, conducted institutional ethics review and approved this study (approval no. 2021/350196).

## Results

A total of 1,179 cases of β-hemolytic streptococcal bloodstream infections were detected during 1999–2021, comprising 309 cases of *S. pyogenes*, 513 cases of *S. agalactiae*, and 354 cases of *S. dysgalactiae*. We excluded 3 Lancefield group C isolates identified as *S. equi* from the analysis. No cases of *S. canis* bloodstream infection were detected. Whole-genome sequencing identified one *S. dysgalactiae* isolate as *S. dysgalactiae* subspecies *dysgalactiae* (*18*), whereas the remaining 353 isolates belonged to the subspecies *equisimilis*.

**Figure 1:**
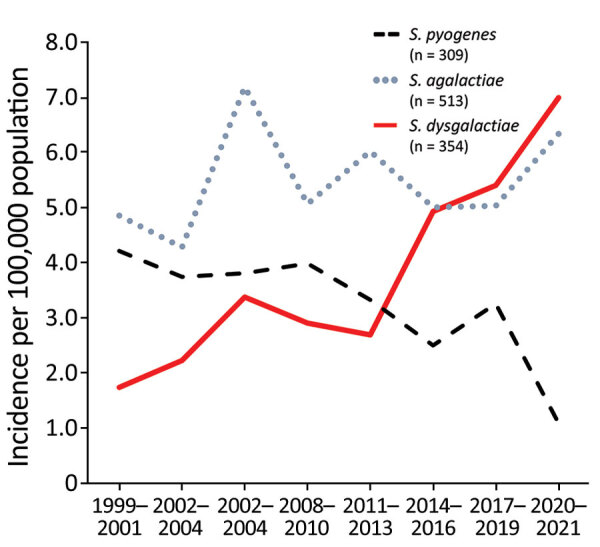
β-hemolytic streptococcal bloodstream infections caused by *Streptococcus pyogenes*, *S. agalactiae*, and *S. dysgalactiae*, western Norway, 1999–2021. We calculated incidence rates for β-hemolytic streptococcal bloodstream infections in Health Region Bergen, Bergen, Norway, in 3-year periods, except 2020–2021. We calculated incidence rates as the total number of cases during each time period, divided by the number of years in the period.

We plotted temporal trends for β-hemolytic streptococcal bloodstream infections (Figure 1). The incidence rate of *S. dysgalactiae* increased significantly from 1.4 to 7.6 (incidence rate ratio [IRR] 1.064, 95% CI 1.045–1.083; p<0.0001) per 100,000 inhabitants, during 1999–2021; by the end of the study period *S. dysgalactiae* had become the leading cause of β-hemolytic streptococcal bloodstream infections. Conversely, *S. pyogenes* displayed decreasing incidence rates (IRR 0.969, 95% CI 0.949–0.988; p = 0.002), whereas we detected no significant temporal change for *S. agalactiae* bloodstream infections (IRR 1.006, 95% CI 0.989–1.024; p = 0.46).

**Figure 2:**
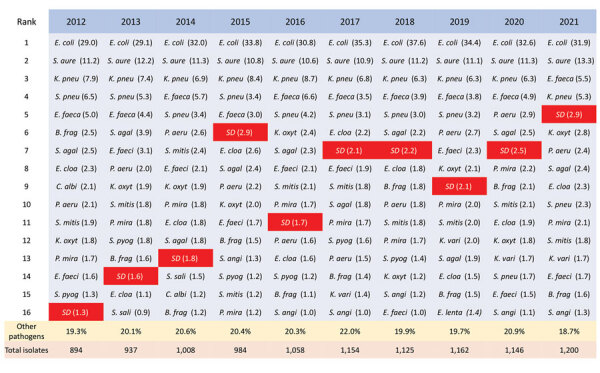
Leading causes of bloodstream infections, western Norway, 2012–2021. We investigated bloodstream infections in Health Region Bergen, Bergen, Norway, and ranked the most common pathogens detected in each year by frequency. Red shading indicates *Streptococcus dysgalactiae* (abbreviated as *SD*). Numbers in parentheses represent the percentage of all identified cases of bloodstream infections for each pathogen. Percentages of other pathogens detected and the total number of bloodstream infections per year are shown across the bottom. *B. frag*, *Bacteroides fragilis*; *C. albi*, *Candida albicans*; *E. cloa*, *Enterobacter cloacae*; *E.coli*, *Escherichia coli*; *E. faeca*, *Enterococcus faecalis*; *E. faeci*, *Enterococcus faecium*; *E. lenta*, *Eggerthella lenta*; *K. oxyt*, *Klebsiella oxytoca*; *K. pneu*, *Klebsiella pneumoniae*; *K. vari*, *Klebsiella variicola*; *P. aeru*, *Pseudomonas aeruginosa*; *P. mira*, *Proteus mirabilis*; *S. agal*, *Streptococcus agalactiae*; *S. angi*, *Streptococcus anginosus*; *S. aure*, *Staphylococcus aureus*; *S. mitis*, *Streptococcus mitis*; *S. pneu*, *Streptococcus pneumoniae*; *S. pyog*, *Streptococcus pyogenes*; *S. sali*, *Streptococcus salivarius*.

Reviewing all bloodstream infections in Health Region Bergen during 2012–2021, we identified 12,346 cases caused by 292 different bacterial species (Appendix Table 1). We excluded 1,678 cases of coagulase-negative staphylococci from our study because we considered these species to be likely skin contaminants. Thus, we included 10,668 cases of bloodstream infection for further analysis. We ranked the most frequent pathogens detected per year and calculated the relative proportion of each pathogen (Figure 2).

The proportion of bloodstream infections caused by *S. dysgalactiae* gradually expanded from 1.3% to 2.9% during the study period, and *S. dysgalactiae* climbed from being the 16th most frequent common in 2012 to the 5th most common in 2021. We noted significantly increasing incidence rates for 3 pathogens: *S. dysgalactiae* (IRR 1.086, 95% CI 1.037–1.137; p<0.0001), *Escherichia coli* (IRR 1.038, 95% CI 1.018–1.058; p = 0.0002), and *Staphylococcus aureus* (IRR 1.031, 95% CI 1.011–1.052; p = 0.002). We found that rates of *S. pneumoniae* bloodstream infections decreased significantly during 2012–2021 (IRR 0.915, 95% CI 0.878–0.953; p<0.0001).

**Figure 3:**
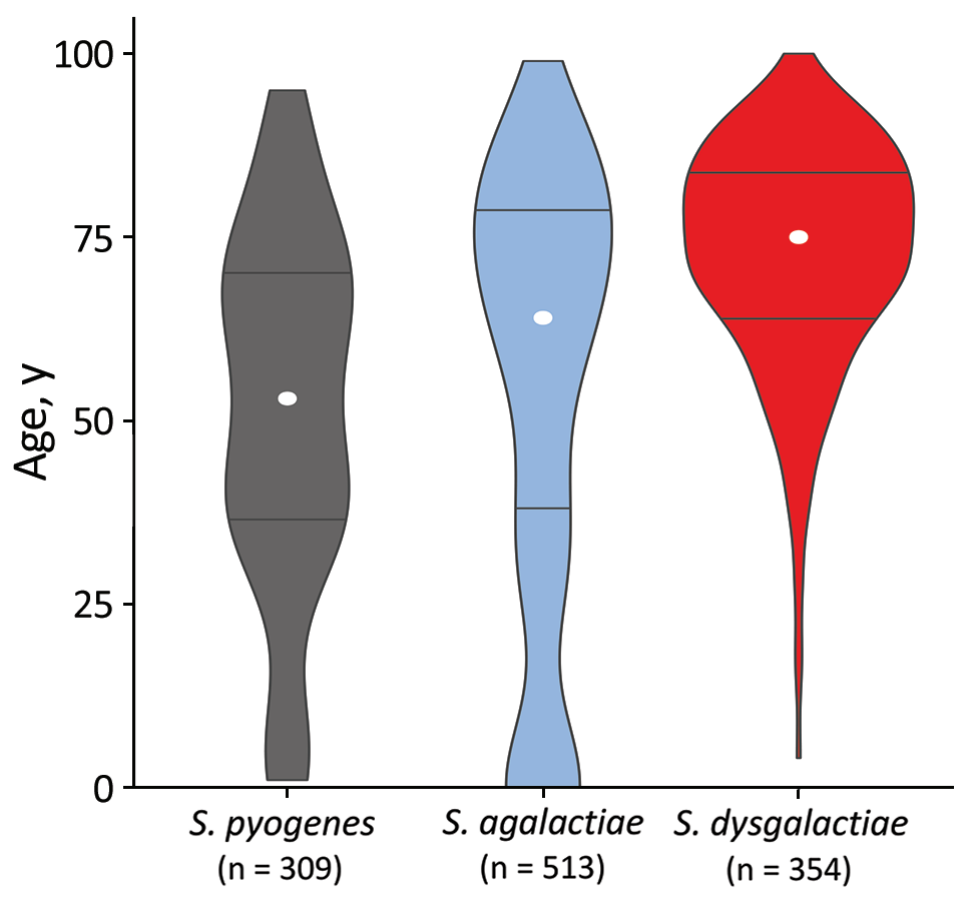
Age distribution for patients with β-hemolytic streptococcal bloodstream infections, western Norway, 1999–2021. The violin plot is based on 1,176 cases of β-hemolytic streptococcal bloodstream infection in Health Region Bergen, Bergen, Norway. The total number of cases is indicated for each species. The width of each figure corresponds to the proportion of patients in that age group. White circles represent the median age and horizontal bars indicate interquartile range.

**Figure 4:**
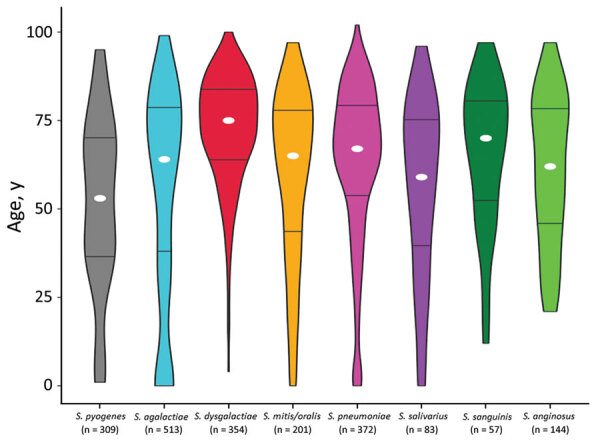
Age distribution for patients with streptococcal bloodstream infections, western Norway, 1999–2021. The violin plot is based on 2,033 cases of bloodstream infection caused by the 8 most common streptococcal species in Health Region Bergen, Bergen, Norway. The total number of cases is indicated for each species. The width of each figure corresponds to the proportion of patients in that age group. White circles indicate the median age and horizontal bars indicate interquartile range.

The 3 β-hemolytic streptococcal species were associated with different profiles of age distribution (Figure 3). *S. dysgalactiae* was intimately linked to higher age (median 75 years, range 4–100 years), and only 2 patients with *S. dysgalactiae* bloodstream infections were <18 years of age. The *S. dysgalactiae* cases were among much older patients than were *S. pyogenes* (median age 53 years, range 1–95 years; p<0.0001) and *S. agalactiae* (median age 64 years, range 0–99 years; p<0.0001) cases. In fact, the median age in the *S. dysgalactiae* group was significantly higher than that for any other major streptococcal pathogen (p<0.0001), including *S. pneumoniae* (median age 67 years), *S. mitis/oralis* (median age 65 years), *S. anginosus* (median age 62 years), *S. salivarius* (median age 61 years), and *S. sanguinis* (median age 71 years) (Figure 4).

**Figure 5:**
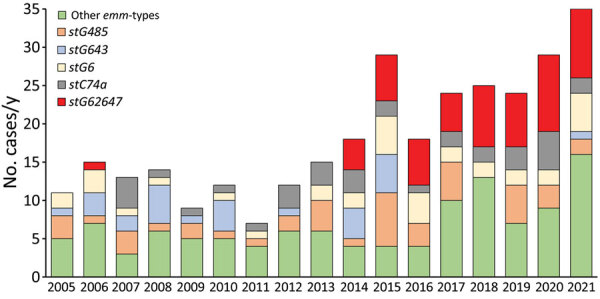
Distribution of the most common *emm*-types among *Streptococcus dysgalactiae* bloodstream infections, western Norway, 2005–2021. We identified most common *emm*-types among 351 *S. dysgalactiae* bloodstream infections in Health Region Bergen, Bergen, Norway. Two isolates were not available for typing, and the *S. dysgalactiae* subspecies *dysgalactiae* isolate could not be typed.

We also assessed *emm*-type distribution among the *S. dysgalactiae* bloodstream infection isolates (Appendix Table 2). We detected high genotypic diversity, comprising 30 different *emm*-types. Genotypic shifts during the study period were evident, and *emm*-type *stG62647* gradually became dominant beginning in 2014 (Figure 5). Inversely, 1 major *emm*-type from early in the study, *stG643*, was infrequently detected after 2015. The remaining *emm*-types displayed annual fluctuations, but we observed no temporal trend. Two isolates were not available for *emm* sequencing.

## Discussion

In this large epidemiologic study of invasive *S. dysgalactiae* bloodstream infections in western Norway, we found that *S. dysgalactiae* is rapidly emerging as a potent pathogen and currently is the fifth most common cause of bloodstream infections in the Bergen health region. *S. dysgalactiae* also appears to have surpassed *S. pyogenes* and *S. agalactiae* as the leading cause of invasive β-hemolytic streptococcal disease in Japan and Finland (*2*,*3*). Moreover, studies from several geographic regions, including Canada, Hungary, Denmark, and Australia, have noted substantial increases in incidence rates for invasive GCGS infections during the past decade (*19*–*22*).

Unfortunately, many larger epidemiologic studies on β-hemolytic streptococcal infections have been hampered by taxonomic imprecision. Several studies report separate incidence curves for group C and group G *Streptococcus*, and some have even exclusively included group G *Streptococcus* (*10*,*11*,*23*,*24*). The lack of species identification is also reflected in the scarce published national surveillance data.

**Figure 6:**
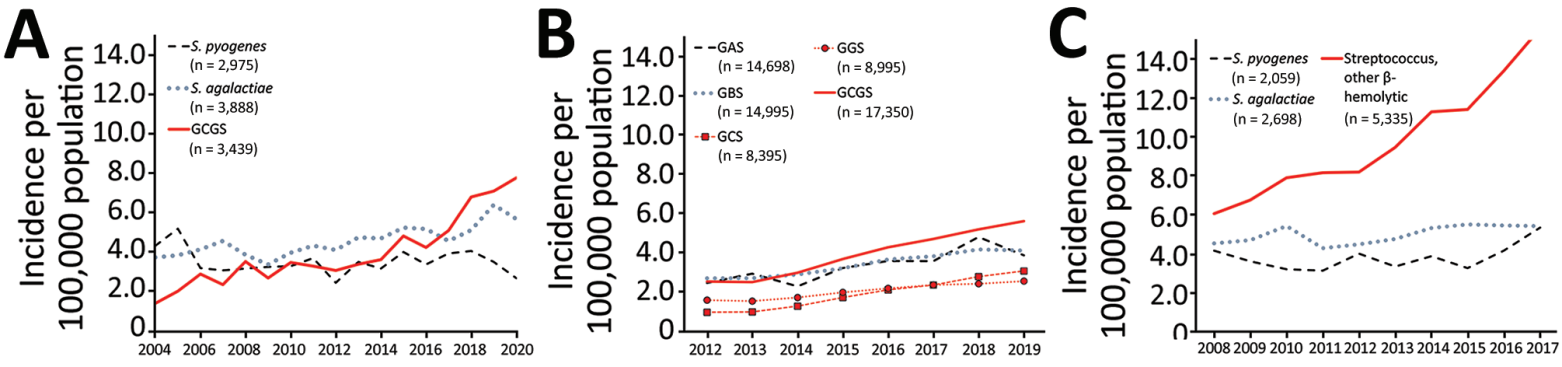
National surveillance data for β-hemolytic streptococcal bloodstream infections in 3 countries: A) Norway, 2004–2020; B) United Kingdom, 2012–2019; C) Finland, 2008–2017. We compiled data from annual surveillance reports published during the given time periods (*6*,*25**–**27*). The 3 countries use different surveillance methods. UK surveillance is based on voluntary reporting from the microbiology laboratories, whereas in Norway and Finland, surveillance data are collected electronically from the laboratories’ information systems. We calculated incidence rates by acquiring contemporary population data from Norway (https://www.ssb.no), the United Kingdom (https://www.ons.gov.uk), and Finland (https://www.stat.fi). We used taxonomic labels that appeared in the original publications, except GCGS, which we constructed for the purpose of this study by combining incidence data for GCS and GGS. GAS, group A *Streptococcus*; GBS, group B *Streptococcus*; GCS, group C *Streptococcus*; GGS, group G *Streptococcus*; GCGS, group C and G *Streptococcus*.

National surveillance on antimicrobial resistance in Norway reports combined annual incidence rates of GCGS bloodstream infections (*25*). In line with our findings, GCGS has been the leading β-hemolytic streptococcal pathogen in Norway since 2017 (Figure 6, panel A). Although not necessarily equivalent to *S. dysgalactiae*, GCGS is a fair estimate of incidence, as demonstrated by the data retrieved from our health region in this study. Nevertheless, the GCGS epithet represents an aggregate of several bacterial species and might easily shroud the magnitude of *S. dysgalactiae* infections from clinicians.

The United Kingdom has monitored β-hemolytic streptococcal bloodstream infections for several decades but tracks group C and group G Streptococcus infections by separate incidence curves (Figure 6, panel B) (*12*). However, that practice can make group C and group G *Streptococcus* appear to be relatively uncommon causes of bloodstream infections. Combining those incidence curves could reveal a completely different picture.

In Finland, the annual incidence rates of invasive *S. pyogenes* and *S. agalactiae* disease are reported separately, whereas *S. dysgalactiae* infections are included in the other β-hemolytic streptococci category (*6*). Of note, the category of other β-hemolytic streptococci greatly expanded during 2008–2017 to become the 4th most common cause of bloodstream infections in Finland (Figure 6, panel C).

Such taxonomic imprecision is detrimental to the surveillance of *S. dysgalactiae* infections. The Lancefield agglutination reaction is a cheap and pragmatic method for sorting β-hemolytic streptococcal isolates but lacks sufficient phylogenetic resolution. To report reliable and comprehensible epidemiologic data, identification to the species level is necessary. MALDI-TOF mass spectrometry is a relatively cheap and efficient alternative, but concerns have been raised about its ability to discern *S. dysgalactiae* from *S. canis* and *S. pyogenes* (*28*). However, modifications and updates to the MALDI-TOF database has largely resolved this issue (*29*). Thus, we propose that future epidemiologic surveillance and publications report the identity of *S. dysgalactiae* to the species level.

The increasing burden of *S. dysgalactiae* disease is likely multifactorial. In line with findings from previous reports, our reports showed this pathogen exhibited a predilection for older persons (*3*,*22*). This preference could be a consequence of immunosenescence but likely also reflects the burden of underlying conditions. Some reports have identified predisposing factors in >90% of the invasive *S. dysgalactiae* cases (*13*,*30*). One report explored risk factors for invasive β-hemolytic streptococcal disease and found the highest average Charlson Comorbidity Index score among patients with GCGS bloodstream infections, including a strong association with diabetes (*22*). In Norway, the estimated life expectancy increased by >5 years during the study period (https://www.ssb.no), and the prevalence of diabetes in the population of Norway has doubled in the past decade (https://www.fhi.no). These developments are projected to continue; thus, the epidemiologic trend for invasive *S. dysgalactiae* disease is unlikely to revert.

Shorter term abrupt changes in incidence rates could be related in part to epidemiologic shifts in circulating *emm*-types. We previously documented the introduction of the *stG62647* *emm*-type into our health region in 2013 (*17*). This *emm*-type appears to have continued to expand and constituted a major contribution to the increasing rates of *S. dysgalactiae* bloodstream infections.

In line with our findings, the *stG62647* genotype appears to have gained a strong foothold in several geographic regions, accounting for 47% of the *S. dysgalactiae* isolates in Germany during 2012–2016 (*15*), 31% in Canada during 2012–2014 (*13*), and 26% in Switzerland during 2006–2015 (*14*). The rapid expansion of this genotype could be related to a lack of herd immunity in the population, but the emergence of a clone with enhanced virulence potential cannot be ruled out. We previously documented a disruption of the streptococcal invasive locus in this genotype, a feature previously associated with increased virulence (*17*,*31*). However, the exact pathogenetic implications of this genetic event have yet to be experimentally elucidated.

Historically, *S. dysgalactiae* has been considered an opportunistic microbe with low pathogenicity, but its potential for severe disease manifestations should not be disregarded. In a large, prospective, multicenter study (*32*), *S. dysgalactiae* was the second most common cause of monomicrobial necrotizing soft tissue infections, and mortality rates for *S. dysgalactiae* cases were even higher than for *S. pyogenes* cases. Moreover, >40% of case-patients experienced septic shock. Recent data from the Swedish Registry of Infective Endocarditis indicated that *S. dysgalactiae* endocarditis was associated with an aggressive clinical course, and rates of embolic events and surgical intervention comparable to endocarditis caused by *Staphylococcus aureus* (*33*). Of note, the *stG62647* genotype was reported as the predominant *emm*-type in both these studies.

Our study is limited by a retrospective design, increasing the risk for taxonomic misclassification. We cannot rule out the possibility that improved taxonomic resolution during the study period could account for some of the increase in the *S. dysgalactiae* disease burden. However, during a thorough review and reconfirmation of all registered cases of β-hemolytic streptococcal infections, including *S. canis* and *S. equi*, we did not identify evidence for such misclassification of the streptococcal isolates. 

Prevention measures taken against the SARS-CoV-2 pandemic had a substantial effect on incidence rates of bacterial diseases, particularly for those caused by airway pathogens (*34*). In line with those effects, epidemiology of bloodstream infections observed in our study might have been influenced to some extent by restrictions during the early pandemic phases, for instance, the rapid decline of *S. pneumoniae* bloodstream infections observed in 2020. Nevertheless, the rising trend of *S. dysgalactiae* predates the pandemic on both a national and international level; this pathogen became the 6th most common cause of bloodstream infections in our region by 2015.

In conclusion, our findings indicate that *S. dysgalactiae* is a potent and common pathogen that should not be overlooked. Enhanced taxonomic precision of this streptococcal species is needed to ensure that epidemiologic data are unambiguous and comprehensible. Moreover, increased attention to *S. dysgalactiae* disease is warranted, and *S. dysgalactiae* should be included in national surveillance programs on equal terms with *S. pyogenes* and *S. agalactiae*.

Appendix Additional information and bacterial strains for *Streptococcus dysgalactiae* and other bloodstream infections, Norway, 2019–2021.
